# Use of Prophylactic Indomethacin in Preterm Infants: A Systematic Review and Meta-Analysis

**DOI:** 10.3389/fped.2022.760029

**Published:** 2022-04-07

**Authors:** Abdulrahman Al-matary, Amani Abu Shaheen, Sameh Abozaid

**Affiliations:** ^1^Neonatology Department, King Fahad Medical City, Riyadh, Saudi Arabia; ^2^Research Center, King Fahad Medical City, Riyadh, Saudi Arabia

**Keywords:** patent ductus arteriosus, intraventricular hemorrhage, prophylactic indomethacin, preterm infants, neonatal outcome

## Abstract

**Background:**

Prophylactic indomethacin has been widely used as an effective intervention for reducing mortalities and morbidities in preterm infants including the cardiopulmonary and neurodevelopmental morbidities such as intraventricular hemorrhage (IVH), but many studies have reported contradictory outcomes regarding its significance. Therefore, we aim to systematically review and meta-analyze the data of prophylactic indomethacin on preterm infants.

**Methods:**

Our systematic search included the following databases: Pubmed, Google Scholar, Scopus, Web of Science, The New York Academy of Medicine (NYAM), Virtual health library (VHL), and the System for Information on Grey Literature in Europe (SIGLE) to include studies that assessed the use of prophylactic indomethacin in preterm infants until 12 August 2021.

**Results:**

The final list of our included studies is comprised of 23 randomized trials and cohort studies. Among all the studies outcomes, significant favorable outcome was lowering the rate of PDA, surgical PDA ligation (*P* < 0.001) and severe IVH (*P* = 0.008) while no significance was recorded with BPD, pulmonary hemorrhage, intraventricular hemorrhage, necrotizing enterocolitis, intestinal perforation, mortality, and length of hospital stay.

**Conclusion:**

Since the meta-analysis results regarding effectiveness of prophylactic indomethacin varied based on the study design particularly with regard to outcomes such as surgical PDA ligation and severe IVH, this warrants the need for more evidence regarding the effectiveness of prophylactic indomethacin in very low birth weight infants.

## Introduction

Many cardiopulmonary and neurologic disabilities have been associated with preterm labor including patent ductus arteriosus (PDA), pulmonary hemorrhage, intracranial hemorrhage, and developmental delay (1–4). Although advances in modern medicine have improved the survival rates of very low birth weight (VLBW) infants, many neurodevelopmental complications are still present due to preterm birth such as blindness, deafness, and cerebral palsy. VLBW infants are at risk of developing intraventricular hemorrhage (IVH) which is usually associated with neurodevelopmental decays when related to the brain parenchyma. IVH grade 3–4 is a major risk factor for the occurrence of these complications in preterm infants (5–8). Although the incidence rate of IVH has been markedly reduced since the 1980s (9, 10), as no or minimal reductions have been recorded recently (11, 12).

Many pre- and postnatal interventions have been reported to effectively treat IVH and reduce its incidence in preterm infants (13). One of these is indomethacin prophylaxis which is better administered within the first 6 h after birth (14–17). Besides, it helps in the closure of ductus arteriosus and therefore, can prevent the complications of PDA such as pulmonary hypertension (14, 15, 18). Its mechanisms of action include prostaglandin synthesis inhibition by inhibiting the cyclooxygenase pathways, reduction of hyperemic responses resulting from cerebrovascular hypoxia and hypercapnia, increasing the blood–brain barrier permeability, and prevention of cerebral perfusion-induced ischemia (19–23). Moreover, it enhances microvascular development in the germinal matrix (24). Perfusion-related factors such as hypoxia, hypercapnia, and hypotension usually develop after birth in VLBW infants (25). Most cases of preterm infants develop IVH within 6–8 h after birth regardless of the gestational age (26). It happens probably due to the increased levels of angiopoietin 2 and vascular endothelial growth factor in the germinal matrix that normally decreases within hours after birth (13).

The results of previously published randomized controlled trials (RCTs) have shown that early administration of indomethacin after birth lowers the incidence of symptomatic PDA and severe IVH as a prophylactic measure (16, 27–29). Although indomethacin administration showed favorable outcomes in reducing IVH incidence, many concerns have arised regarding its effect on cerebral perfusion (30, 31). The rates of mortalities, bronchopulmonary dysplasia (BPD), or long-term neurodevelopmental decays reportedly seem to have been not affected. A previously published large RCT advised against using indomethacin as a prophylactic agent (15). Although the study showed favorable outcomes in terms of reducing incidence rates of PDA, PDA ligation, IVH, and pulmonary hemorrhage, no improvement regarding the incidence of death and neurodevelopmental disorders rates has been found. Therefore, in this systematic review, we aim to analyze the data of previously published investigations on the use of prophylactic indomethacin in preterm infants.

## Materials and Methods

### Search Strategy and Study Selection

In accordance with the Preferred Reporting Items for Systematic Review and Meta-analyses statement (PRISMA) recommendations, we performed this systematic review and meta-analysis (32). A systematic electronic database search was conducted for relevant studies published, from inception until 12 August 2021, in seven databases: Pubmed, Google Scholar, Scopus, Web of Science, The New York Academy of Medicine (NYAM), Virtual health library (VHL), and the System for Information on Grey Literature in Europe (SIGLE). The search process was conducted using keywords, medical subject (MeSH) terms, and publication types based on the PICO framework (participants, comparison, intervention, and outcomes). Participants were any preterm infants, the intervention was the prophylactic indomethacin, the comparison was placebo or no treatment groups, and all possible outcomes were included. The systematic search was followed by a manual search in references of the included papers to include missed papers (33).

We included all original studies that assessed the use of prophylactic indomethacin in preterm infants. Papers were excluded if there were one of the following exclusion criteria: (i) non-original studies; (ii) articles in non-English language; (iii) *in vitro* or animal studies; (iv) data duplication, overlapping or unreliably extracted or incomplete data; and (v) abstract only articles, reviews, thesis, books, conference papers, or articles without available full texts (conferences, editorials, author response, letters, and comments). The title and abstract screening were performed by four independent reviewers. Furthermore, three independent reviewers performed full-text screening to ensure the inclusion of relevant papers in our systematic review. Any disagreement was done by discussion and consulting the senior member when necessary.

### Data Extraction

Two authors made the pilot extraction of a few papers for building the data extraction sheet. The data extraction sheet included: patient’s characteristics, and outcomes. Two authors extracted the data and was reviewed by a third reviewer when necessary. If a disagreement occurred, a senior author was consulted.

### Statistical Analysis

All data were analyzed using Comprehensive Meta-analysis Software Version 3.0, odds ratios (OR) and Standardized mean difference (SDM) outcomes were calculated. The corresponding 95% confidence intervals (CI) of pooled effect size were calculated using a fixed-effects or random-effects, according to heterogeneity level. Heterogeneity was assessed with Q statistics and I^2^ test.

The publication bias was assessed using Egger’s regression test (34, 35) and represented graphically by Begg’s funnel plot (36). when there were 10 or more studies/effect sizes. Egger’s regression test *P*-value <0.10 was considered significant. Whenever publication bias was found, the trim and fill method of Duvall and Tweedie was applied (37) to add studies that appeared to be missing to enhance the symmetry.

## Results

### Search Results

**FIGURE 1:**
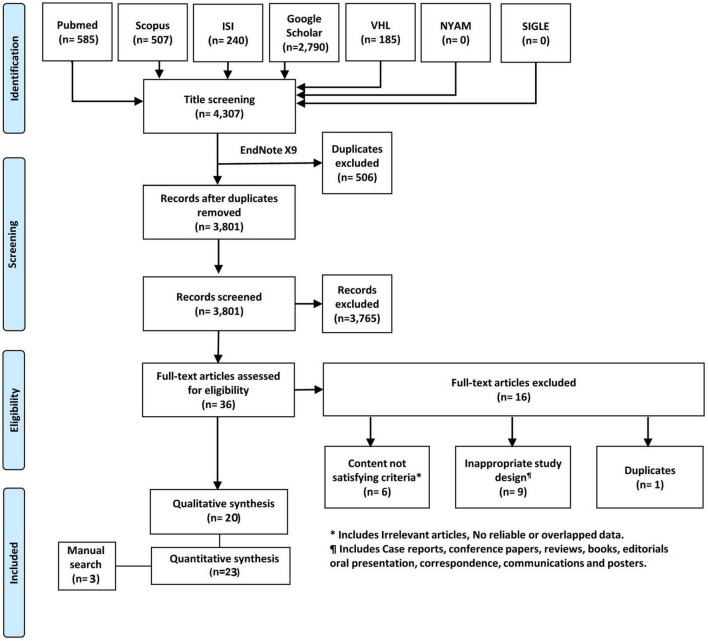
PRISMA flowchart of the search and screening process.

We identified 3,801 records after excluding of 506 duplicates by using Endnote software version X9. Title and abstract screening resulted in 36 records for further full-text screening. The later yielded 20 eligible papers for inclusion in our study. Three papers were added after performing manual search trials. Finally, we included 23 studies for this systematic review and meta-analysis (Figure 1).

### Study Characteristics

Out of the 23 included studies; 15 were randomized controlled trials and the remaining eight were cohort in design. The sample size of the included studies was highly variable ranging from 19 and as high as 34,602 pre-term infants. The average mean age in all reported treatment group and control group was 27 weeks (ranging from 26 to 28 weeks). Table 1 shows the main characteristics of the included studies (15, 17, 27–29, 38–55).

**TABLE 1:** Characteristics of the included studies.

Author Year	Design	Sample size	Gestational age						Birth weight						Male				Aim	Main conclusion(s)
			Treatment group			Control group			Treatment group			Control group			Treatment group		Control group			
		
			Total	Mean	SD	Total	Mean	SD	Total	Mean	SD	Total	Mean	SD	Event	Total	Event	Total		
Bada et al. (28)	RCT	141	71	28	2.2	70	28	2.6	71	1,103	253	70	1,074	265	37	71	26	70	To determine the efficacy of indomethacin in preventing periventricular-intraventricular hemorrhage (PV-IVH)	indomethacin prophylaxis reduced the relative risk of grades 2 to 4 PV-IVH and severe PV-IVH, but other perinatal variables contributed significantly to the overall risk of PV-IVH
Bandstra et al. (27)	RCT	199	99	29	2.3	100	29.3	2.1	99	970	174	100	970	183	51	99	43	100	To assess the impact of early prophylactic use of intravenous indomethacin on the incidence and severity of periventricular-intraventricular hemorrhage and patent ductus arteriosus in 199 oxygen-requiring premature infants	Early prophylactic indomethacm initiated within 12 h of delivery is effective in reducing the incidence of intraventnicular hemorrhage as well as clinically significant patent ductus arteniosus in very low birth weight premature infants
Jensen et al. (38)	Cohort	7,831	2,587	25.9	1.5	5,244	26.7	1.6	2,587	777	197	5,244	913	246	1,270	2,587	2,744	5,244	To assess the association between prophylactic indomethacin and bronchopulmonary dysplasia (BPD) in a recent, large cohort of extremely preterm infants	Prophylactic indomethacin was not associated with either reduced or increased risk for BPD or death
Laughon et al. (39)	Cohort	34,602	–	–	–	–	–	–	–	–	–	–	–	–	3,293	6,189	15,406	28,413	To describe the current use of treatments to prevent or treat patent ductus arteriosus (PDA) in preterm infants, examine the association between different treatment strategies and neonatal outcomes and review the variation in these practices between centers	Indomethacin use for intraventricular hemorrhage prevention and/or treatment of a PDA is common, but the selection of infants for treatment, and the decision of when and how to treat vary widely between centers. Our findings suggest the need for randomized, placebo-controlled trials of the effect of treatment of the PDA in preterm infants
Liebowitz et al. (40)	Cohort	397	247	26.1	1.2	150	26	1.2	247	813	197	150	802	200	117	247	90	150	To determine whether prophylactic indomethacin (prophylactic indomethacin treatment) has more or less morbidity than delayed conservative management of the moderate-to-large patent ductus arteriosus (PDA)	
Maruyama et al. (41)	RCT	19	–	–	–	–	–	–	–	–	–	–	–	–	–	–	–	–	To investigate the effects of prophylactic low-dose indomethacin on renal and intestinal blood flow	Prophylactic low-dose indomethacin increases the diastolic blood flow in the RAand SMAvia a reduction in the ductal shunt volume, with no change in the regional vascular resistance
Mirza et al. (42)	Cohort	868	868	26.36	1.97	–	–	–	868	864.82	210.84	–	–	–	431	868	–	–	To test the hypothesis that administration of indomethacin prophylaxis before 6 hours of life results in a lower incidence of intraventricular hemorrhage (IVH) compared with administration after 6 h of life, and that the effects of early prophylaxis depend on gestational age (GA) and sex in very low birth weight infants (birth weight <1,250 g)	Prophylactic indomethacin administered before 6 h of life is not associated with lower incidence of IVH
Narayanan et al. (43)	Cohort	300	130	25.5	1.1	170	25.5	1.1	130	798	172	170	803	180	68	130	87	170	To examine the role of prophylactic indomethacin in producing permanent DA closure and the mechanism by which this occurs	Prophylactic indomethacin improved the rate of permanent ductus closure by increasing the degree of initial constriction. Prophylactic indomethacin did not affect the remodeling process, nor did it alter the inverse relationship between infant maturity and subsequent reopening. Even when managed with prophylactic indomethacin, the rate of ductus reopening remained unacceptably high in the most immature infants
Nelin et al. (44)	Cohort	671	–	–	–	–	–	–	–	–	–	–	–	–	–	–	–	–	To determine whether PI use in a contemporary cohort of EP infants admitted to an all-referral NICU continues to be associated with beneficial outcomes	PI administration was associated with improved survival in EP infants referred to a level IV Children’s Hospital NICU
Schmidt et al. (15)	RCT	1,202	601	25.9	1.8	601	26	1.9	601	782	131	601	783	130	309	601	306	601	To determine whether the prophylactic administration of indomethacin improves survival without neurosensory impairment in extremely-low-birth-weight infants (those with birth weights below 1,000 g)	In extremely-low-birth-weight infants, prophylaxis with indomethacin does not improve the rate of survival without neurosensory impairment at 18 months, despite the fact that it reduces the frequency of patent ductus arteriosus and severe periventricular and intraventricular hemorrhage
Stavel et al. (45)	Cohort	4,268	–	–	–	–	–	–	–	–	–	–	–	–	244	498	1,855	3,770	To determine the effect of concomitant administration of prophylactic indomethacin and early enteral feeds on the risk of spontaneous intestinal perforation (SIP) in extremely low-birth-weight (ELBW) infants, and to describe the variation in prophylactic indomethacin use in Canada	Prophylactic indomethacin was associated with increased odds of SIP independently from early feeding in this cohort; however, early enteral feeding was not associated with SIP. Marked variation in the use of prophylactic indomethacin was identified
Couser et al. (46)	RCT	99	43	26.4	1.6	47	26.4	1.8	43	915	209	47	879	202	25	43	22	47	To determine whether a course of low-dose indomethacin therapy, when initiated within 24 h of birth, would decrease ductal shunting in premature infants who received prophylactic surfactant in the delivery room	The prophylactic use of low doses of indomethacin, when initiated in the first 24 h of life in low birth weight infants who receive prophylactic surfactant in the delivery room, decreases the incidence of left-to-right shunting at the level of the ductus arteriosus
Hanigan et al. (47)	RCT	122	56	30.00	0.3	55	29.7	0.3	56	1,138	31.7	1,153	32.1		30	56	29	55	To test the null hypothesis that the prophylactic administration of indomethacin would not be associated with a significant reduction in the incidence of PVH-IVH	Prophylactic administration of intravenous indomethacin for the prevention of PVH-IVH cannot be recommended for infants <1,000 g. In preterm infants between I000 and 1,500 g birth weight, indomethacin significantly reduced the incidence of PVH-IVH
Krueger et al. (48)	RCT	32	15	29.4	0.4	17	28.9	0.4	15	1,126	52	17	1,111	47	10	15	8	17	To determine the efficacy of indomethacin to prevent the occurrence of symptomatic patent ductus arteriosus (PDA)	Results indicate that the use of prophylactic indomethacin is beneficial in prevention of symptomatic PDA
Yaseen et al. (49)	RCT	27	14	30.3	2.5	13	29.1	3.1	14	1,320	350	13	1,230	360	8	14	7	13	To evaluate the oxygenation, and surfactant requirements in preterm low birth weight infants receiving early indomethacin administration	Early indomethacin administration increases oxygen and surfactant requirement
Vincer et al. (50)	RCT	30	15	28.0	25-34	15	29.0	26-36	15	940	700–1,480	15	970	520–1,480	8	15	8	15	To test the efficacy of early intravenous indomethacin therapy in preventing chronic pulmonary disease of prematurity	Data suggests that caution must be exercised with early use of indomethacin
Ment et al. (51)	RCT	48	24	28.7	1.92	24	28.5	2.20	24	1,010	172	24	1,015	156	–	–	–	–	To examine the use of indomethacin to prevent GMH/IVH in very low birth weight neonates.	Indomethacin should only be used investigationally for the prevention of GMH/IVH, with particular attention to long-term neurodevelopmental outcome and the incidence of severe IVH
Ment et al. (52)	RCT	36	19	28.2	1.9	17	2,813	2.0	19	950	152	17	927	175	10	19	10	17	To determine whether a low dose of indomethacin would prevent germinal matrix or intraventricular hemorrhage and permit adequate urinary output	Ductal status appeared unrelated to the development of germinal matrix or intraventricular hemorrhage
Ment et al. (16)	RCT	61	–	–	–	–	–	–	–	–	–	–	–	–	–	–	–	–	To test if indomethacin (0.1 mg/kg given intravenously at 6–12 postnatal hours and every 24 h for two more doses) would prevent extension of intraventricular hemorrhage	In very low birth weight infants with low grade intraventricular hemorrhage within the first 6 postnatal hours, prophylactic indomethacin promotes closure of the patent ductus arteriosus and is not associated with adverse events, but does not affect the events leading to parenchymal involvement of intracranial hemorrhage
Nair et al. (53)	RCT	115	56	27.8	1.2	59	27.9	1.4	56	989.5	93.5	59	995	83.6	–	–	–	–	To study the efficacy and complications of low dose indomethacin in the reduction of major intraventricular hemorrhage (IVH) in very low birth weight (VLBW) babies.	Indomethacin prophylaxis did not confer protection against IVH in very low birth weight babies. Instead it showed an increase in the risk of IVH, other bleeding episodes and chronic lung disease
Rennie et al. (54)	RCT	50	24	28	2.3	26	29	2.0	24	1,214	323	26	1,330	326	13	24	18	26	To temporally relate plasma 6-ketoprostaglandin Fla, indomethacin concentrations, and clinical response in a group of low birthweight infants receiving intensive care	There was no significant difference in the incidence of intraventricular hemorrhage, days of treatment with oxygen or ventilation, or mortality between the two groups
Mahony et al. (55)	RCT	104	51	28.0	1.5	53	28.0	1.6	51	1,020.0	158.0	53	989.0	162.0	21	51	32	53	To investigate the optimal timing for treatment of small premature infants using indomethacin therapy on the first day of life	Although treatment with indomethacin on the first day of life appears to be safe, there is little advantage to its use in centers where the incidence of large shunts through a patent ductus arteriosus is relatively low

*SD, standard deviation; RCT, randomized controlled trial.*

### Publication Bias

With regard to articles with a cohort study design, no publication bias was found in the studies relating to the outcome of death (*P* = 0.852) using Begg’s adjusted rank correlation test. Publication bias related to bronchopulmonary dysplasia, severe intraventricular hemorrhage, necrotizing enterocolitis and surgical PDA ligation was not assessed owing to few number of studies.

**FIGURE 2:**
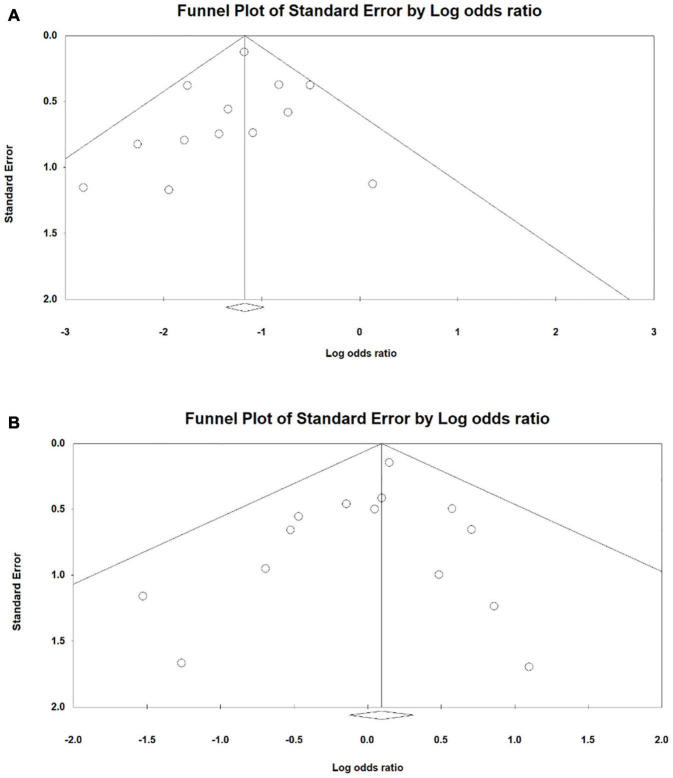
Publication bias among randomized controlled trial studies for the outcome **(A)** patent ductus arteriosus **(B)** death.

Regarding publication bias among RCT studies, overall no publication bias was found in the studies. Regarding PDA, no publication bias was found in the studies (*P* = 0.524) using Egger’s test (Figure 2A). No publication bias was found in studies (*P* = 0.458) using Begg’s adjusted rank correlation test with regard to severe interventricular hemorrhage. Regarding necrotizing enterocolitis, no publication bias was found in studies (*P* = 0.652) using Begg’s adjusted rank correlation test. With regard to death, no publication bias was found in the studies (*P* = 0.394) using Egger’s test (Figure 2B). Publication bias related to bronchopulmonary dysplasia, intraventricular hemorrhage, pulmonary hemorrhage, intestinal perforation, surgical PDA ligation and hospitalization days were not assessed owing to few studies.

**FIGURE 3:**
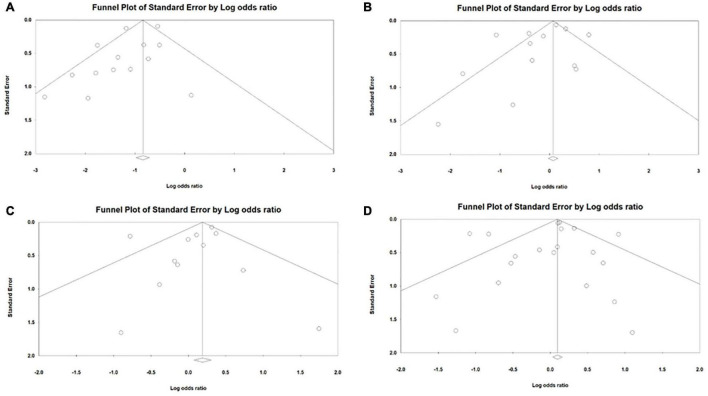
Publication bias among cohort and randomized controlled trial study designs for the outcome **(A)** patent ductus arteriosus **(B)** interventricular hemorrhage **(C)** necrotizing enterocolitis **(D)** death.

With regard to the publication bias in both cohort and RCT studies, in general no publication bias was seen with the exception of patent ductus arteriosus where publication bias was found in the studies (*P* = 0.083) using Egger’s test (Figure 3A). No publication bias was found related to bronchopulmonary dysplasia in studies (*P* = 0.543) using Begg’s adjusted rank correlation test. With regard to intraventricular hemorrhage, no publication bias was found in studies (*P* = 0.348) using Begg’s adjusted rank correlation test. For severe interventricular hemorrhage as well, no publication bias was found in studies (*P* = 0.217) using Egger’s test (Figure 3B). Regarding necrotizing enterocolitis, no publication bias was found in studies (*P* = 0.364) using Egger’s test (Figure 3C). With regard to death, no publication bias was found in studies (*P* = 0.449) using Egger’s test (Figure 3D). Using Begg’s adjusted rank correlation test, no publication bias was found in studies (*P* = 0.176) with regard to surgical PDA ligation. Publication bias related to pulmonary hemorrhage, intestinal perforation and hospitalization days was not assessed owing to few studies.

### Meta-Analysis of Outcomes

#### Bronchopulmonary Dysplasia

**FIGURE 4:**
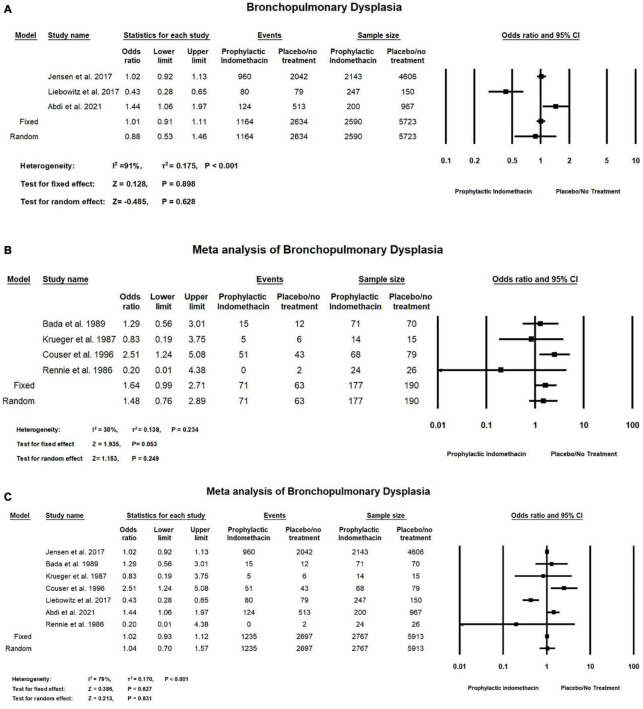
Meta-analysis of bronchopulmonary dysplasia from **(A)** cohort studies, **(B)** RCT studies, **(C)** combination of cohort and RCT studies.

In the meta-analysis of cohort studies, no significant difference was seen between the group of infants given prophylactic doses of indomethacin and the placebo or no treatment group with regard to the rates of bronchopulmonary dysplasia (OR = 0.88; 95% CI = 0.53–1.46; *P*-value = 0.628). There was high significant heterogeneity among the included studies (I^2^ = 91%; *P*-value < 0.001) (Figure 4A).

Meta-analysis of RCT studies shows there was no significant difference between the group of infants with prophylactic doses of indomethacin and the group of placebo or no treatment with regard to the rates of bronchopulmonary dysplasia (OR = 1.64; 95% CI = 0.99–2.71; *P*-value = 0.053). There was no significant heterogeneity among the included studies (I^2^ = 30%; *P*-value = 0.234) (Figure 4B).

In the combined meta-analysis of cohort and RCT studies, there was no significant difference between the prophylactic indomethacin group and the placebo or no treatment group regarding the rates of bronchopulmonary dysplasia (OR = 1.04; 95% CI = 0.70–1.57; *P*-value = 0.831). There was high significant heterogeneity among the included studies (I^2^ = 79%; *P*-value < 0.001) (Figure 4C).

#### Patent Ductus Arteriosus

**FIGURE 5:**
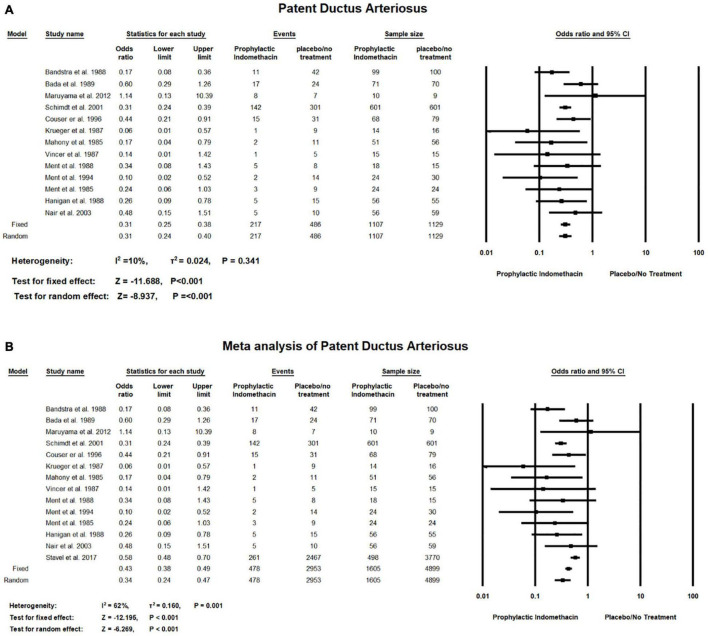
Meta-analysis of patent ductus arteriosus from **(A)** RCT studies, **(B)** combination of cohort and RCT studies.

Meta-analysis of RCT studies shows infants given prophylactic doses of indomethacin have significantly lower rates of PDA compared to those who did not (OR = 0.31; 95% CI = 0.25–0.38; *P*-value < 0.001). There was no significant heterogeneity among the included studies (I^2^ = 10%; *P*-value = 0.341) (Figure 5A).

Combined meta-analysis of cohort and RCT studies shows infants given prophylactic doses of indomethacin have significantly lower rates of Patent Ductus Arteriosus compared to those who did not (OR = 0.34; 95% CI = 0.24–0.47; *P*-value < 0.001). However, there was medium significant heterogeneity among the included studies (I^2^ = 62%; *P*-value = 0.001) (Figure 5B).

#### Surgical PDA Ligation

**FIGURE 6:**
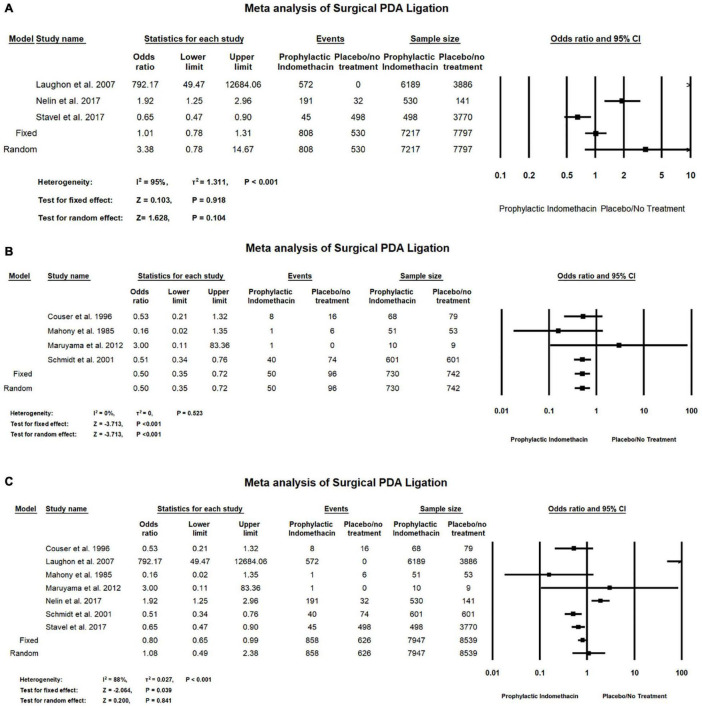
Meta-analysis of surgical PDA ligation from **(A)** cohort studies, **(B)** RCT studies, **(C)** combination of cohort and RCT studies.

In the meta-analysis of cohort studies, there was no significant differences between the group of infants given prophylactic doses of indomethacin and placebo or no treatment group with regard to the rates of surgical PDA ligation (OR = 3.84; 95% CI = 0.78–14.67; *P*-value = 0.104). There was high significant heterogeneity among the included studies (I^2^ = 95%; *P*-value < 0.001) (Figure 6A).

Meta-analysis of RCT studies shows infants with prophylactic doses of indomethacin have significantly lower rates of surgical PDA ligation compared to those who did not (OR = 0.50; 95% CI = 0.35–0.72; *P*-value < 0.001). There was no significant heterogeneity among the included studies (I^2^ = 0%; *P*-value = 0.523) (Figure 6B).

Combined meta-analysis of cohort and RCT studies shows there was no significant differences between the group of infants given prophylactic doses of indomethacin and the infants in the placebo or no treatment group with regard to the rates of necrotizing enterocolitis (OR = 1.10; 95% CI = 0.79–1.52; *P*-value = 0.571). There was no significant heterogeneity among the included studies (I2 = 0%; *P*-value = 0.825) (Figure 6C).

#### Pulmonary Hemorrhage

**FIGURE 7:**
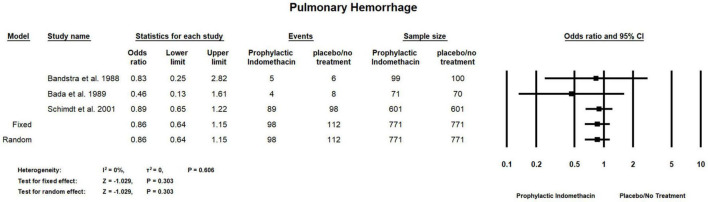
Meta-analysis of pulmonary hemorrhage from RCT studies.

Meta-analysis of RCT studies shows there was no significant differences between the group of infants given prophylactic doses of indomethacin and placebo or no treatment group regarding the rates of pulmonary hemorrhage (OR = 0.86; 95% CI = 0.64–1.15; *P*-value = 0.303). There was no significant heterogeneity among the included studies (I^2^ = 0%; *P*-value = 0.606) (Figure 7).

#### Intraventricular Hemorrhage

**FIGURE 8:**
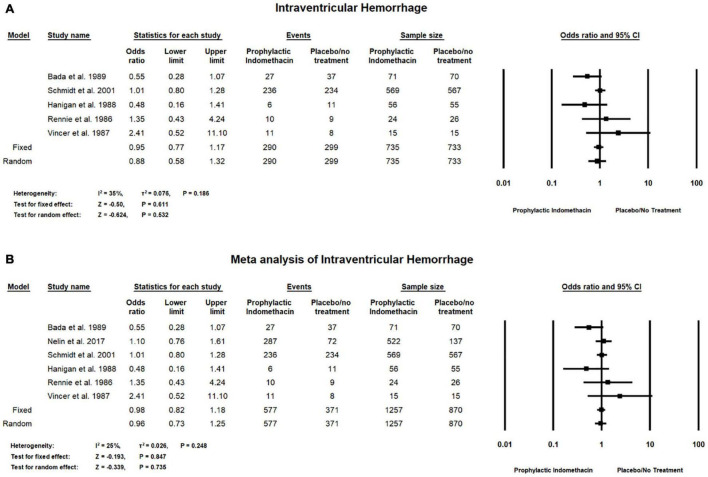
Meta-analysis of intraventricular hemorrhage from **(A)** RCT studies, **(B)** combination of cohort and RCT studies.

Meta-analysis of RCT studies shows there was no significant differences between the group of infants given prophylactic doses of indomethacin and the placebo or no treatment group with regard to the rates of intraventricular hemorrhage (OR = 0.88; 95% CI = 0.58–1.32; *P*-value = 0.532). There was low heterogeneity among the included studies (I^2^ = 35%; *P*-value = 0.186) (Figure 8A).

Combined meta-analysis of cohort and RCT studies shows there was no significant differences between the group with infants given prophylactic doses of indomethacin and placebo or no treatment group with regard to the rates of intraventricular hemorrhage (OR = 0.96; 95% CI = 0.73–1.25; *P*-value = 0.735). There was low heterogeneity among the included studies (I^2^ = 25%; *P*-value = 0.248) (Figure 8B).

#### Severe Intraventricular Hemorrhage

**FIGURE 9:**
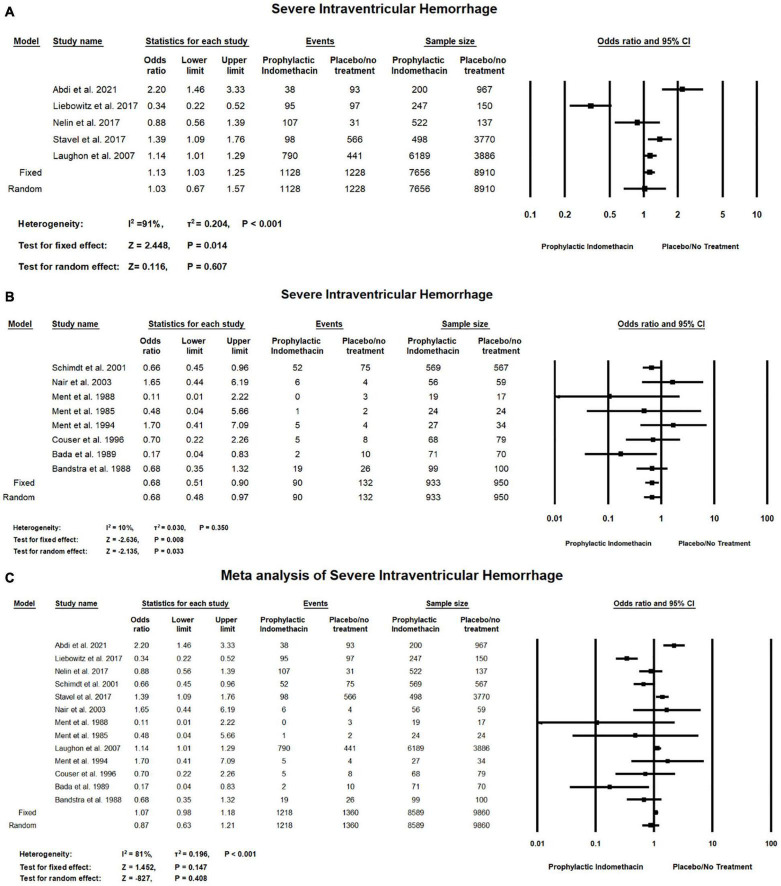
Meta-analysis of severe intraventricular hemorrhage from **(A)** cohort studies, **(B)** RCT studies, **(C)** combination of cohort and RCT studies.

In the meta-analysis of cohort studies, no significant difference was found between the group of infants given prophylactic doses of indomethacin and the placebo or no treatment group regarding the rates of severe intraventricular hemorrhage (OR = 1.03; 95% CI = 0.67–1.57; *P*-value = 0.607). There was high significant heterogeneity among the included studies (I^2^ = 91%; *P*-value < 0.001) (Figure 9A).

For meta-analysis of RCT studies, as seen in Figure 9B, infants with prophylactic doses of indomethacin have significantly lower rates of severe intraventricular hemorrhage compared to those who did not (OR = 0.68; 95% CI = 0.51–0.90; *P*-value = 0.008). There was no significant heterogeneity among the included studies (I^2^ = 10%; *P*-value = 0.350).

In the combined meta-analysis of cohort and RCT studies, no significant differences between the group of infants given prophylactic doses of indomethacin and the placebo or no treatment group regarding the rates of severe intraventricular hemorrhage (OR = 0.87; 95% CI = 0.63–1.21; *P*-value = 0.408). However, there was high significant heterogeneity among the included studies (I^2^ = 81%; *P*-value < 0.001) (Figure 9C).

#### Necrotizing Enterocolitis

**FIGURE 10:**
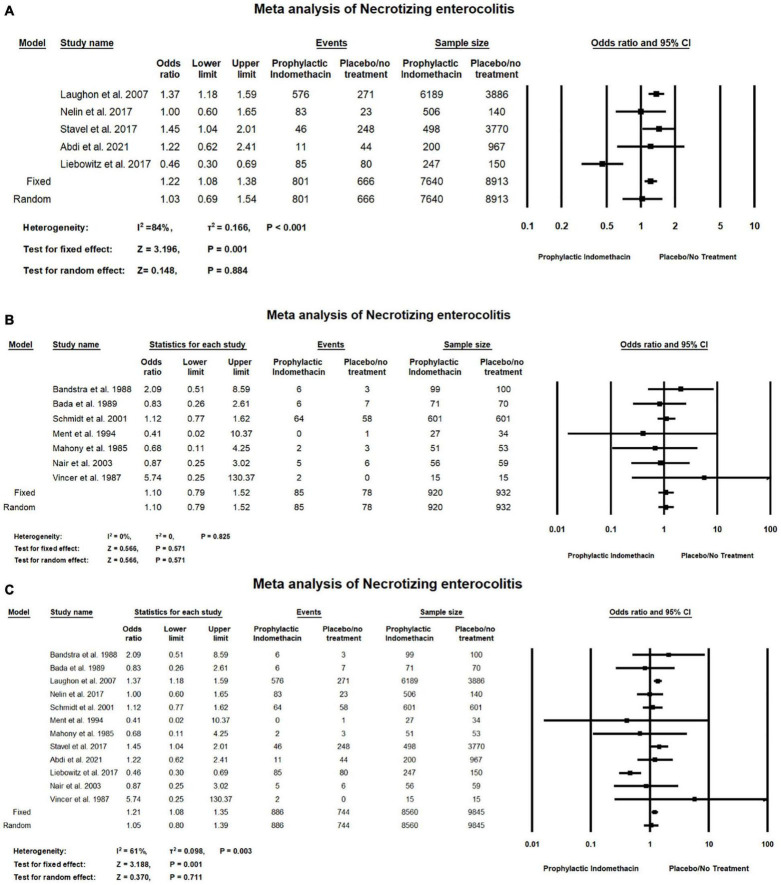
Meta-analysis of necrotizing enterocolitis from **(A)** cohort studies **(B)** RCT studies **(C)** combination of cohort and RCT studies.

In the meta-analysis of cohort studies, regarding the rate of necrotizing enterocolitis, there was no significant differences between the group of infants with prophylactic doses of indomethacin and the infants in the placebo or no treatment group (OR = 1.03; 95% CI = 0.69–1.54; *P*-value = 0.884). There was high significant heterogeneity among the included studies (I^2^ = 84%; *P*-value < 0.001) (Figure 10A).

Meta-analysis of RCTs shows there was no significant difference between the group of infants given prophylactic doses of indomethacin and the infants in the placebo or no treatment group with regard to the rates of necrotizing enterocolitis (OR = 1.10; 95% CI = 0.79–1.52; *P*-value = 0.571). There was no significant heterogeneity among the included studies (I2 = 0%; *P*-value = 0.825) (Figure 10B).

Combined meta-analysis of cohort and RCT studies shows there was no significant differences between the group of infants with prophylactic doses of indomethacin and the infants in the placebo or no treatment group regarding the rates of necrotizing enterocolitis (OR = 1.05; 95% CI = 0.80–1.39; *P*-value = 0.711). However, there was medium significant heterogeneity among the included studies (I^2^ = 61%; *P*-value = 0.003) (Figure 10C).

#### Intestinal Perforation

**FIGURE 11:**
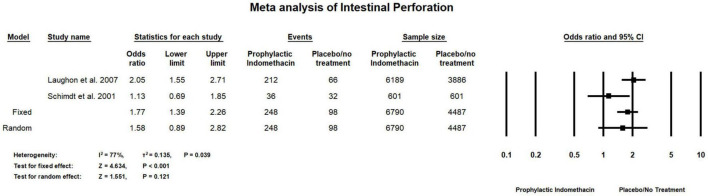
Meta-analysis of intestinal perforation from both cohort and RCT studies.

Combined meta-analysis of cohort and RCT studies, shows there was no significant differences between the group of infants given prophylactic doses of indomethacin and the infants in the placebo or no treatment group with regard to the rates of intestinal perforation (OR = 1.58; 95% CI = 0.89–2.82; *P*-value = 0.121). However, there was high significant heterogeneity among the included studies (I^2^ = 77%; *P*-value = 0.039) (Figure 11).

#### Hospitalization Days

**FIGURE 12:**
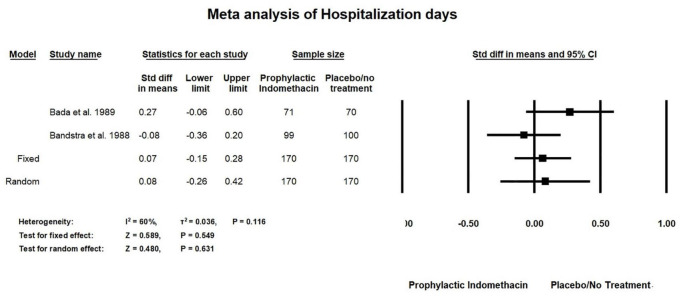
Meta-analysis of hospitalization days from RCT studies.

Meta-analysis of RCT studies shows that two studies with 340 patients were included in the analyses of hospitalization days. On comparing this outcome among the prophylactic indomethacin and placebo/no treatment groups, there was no statistically significant difference for hospitalization days (SMD = 0.08; 95% CI = -0.26: 42; *P*-value = 0.631). There was a medium significant heterogeneity in the analysis of hospitalization days (I^2^ = 60%; *P*-value = 0.116) (Figure 12).

#### Death

**FIGURE 13:**
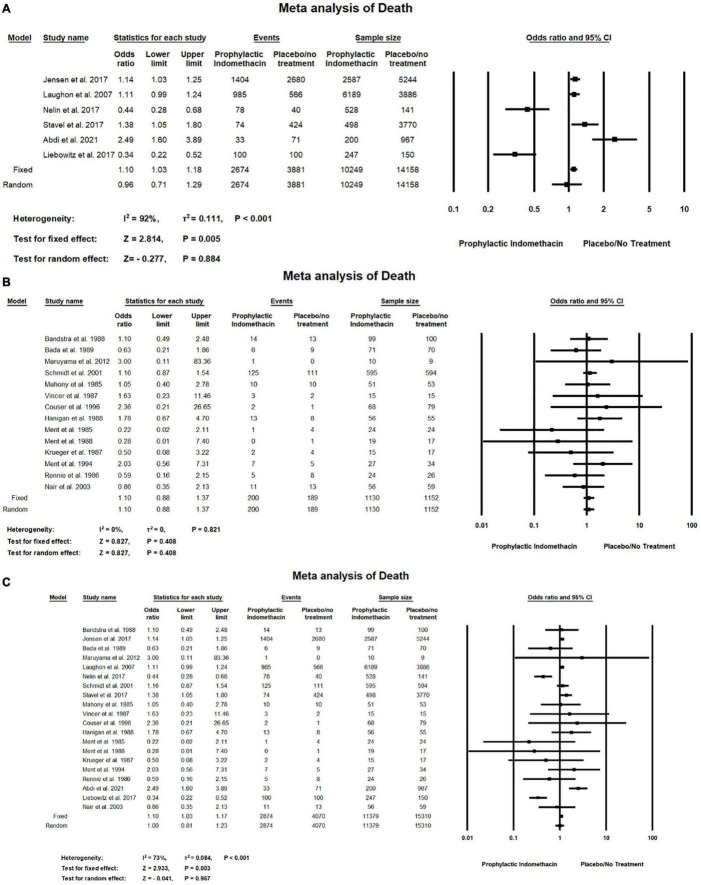
Meta-analysis of death from **(A)** cohort studies, **(B)** RCT studies, **(C)** combination of cohort and RCT studies.

In the meta-analysis of cohort studies, Figure 13A shows there was no significant differences between the group of infants given prophylactic doses of indomethacin and the placebo or no treatment group with regard to the rates of death (OR = 0.96; 95% CI = 0.71–1.29; *P*-value = 0.884). There was high significant heterogeneity among the included studies (I^2^ = 92%; *P*-value < 0.001).

Meta-analysis of RCT studies shows there was no significant differences between the group of infants given prophylactic doses of indomethacin and the placebo or no treatment group regarding the rates of death (OR = 1.10; 95% CI = 0.88–1.37; *P*-value = 0.408). There was no significant heterogeneity among the included studies (I^2^ = 0%; *P*-value = 0.821) (Figure 13B).

Combined meta-analysis cohort and RCT studies shows there was no significant differences between the group of infants given prophylactic doses of indomethacin and the group of placebo or no treatment regarding the rates of death (OR = 1.00; 95% CI = 0.81–1.23; *P*-value = 0.967). However, there was high significant heterogeneity among the included studies (I^2^ = 73%; *P*-value < 0.001) (Figure 13C).

## Discussion

In this study, we have included 23 studies from the systematic and manual search to be analyzed to study indomethacin as a prophylactic measure in pre-term infants from many aspects including bronchopulmonary dysplasia, patent ductus arteriosus, pulmonary hemorrhage, intraventricular hemorrhage, severe intraventricular hemorrhage, necrotizing enterocolitis, intestinal perforation, death, hospitalization days, and surgical ligation of PDA.

The analyzed data showed a varied heterogeneity in some outcomes which is probably due to the difference in study designs, the different dosages of indomethacin injection, and outcome definition between studies. Moreover, it is important to note that this meta-analysis is fundamentally different from prior ones, in that data from both randomized trials and retrospective cohort studies are included in the present analyses and is likely to be the dominant factor for differences in results.

As for the cardiopulmonary outcomes, our meta-analysis of RCT studies and combined meta-analysis of RCT and cohort studies showed that prophylactic indomethacin administration in infants significantly lowers the rates of PDA formation (*P*-value < 0.001) and no significant heterogeneity was estimated (I^2^ = 10%; *P*-value = 0.341) in case of the included RCT studies while medium significant heterogeneity was found in the combined analysis of RCT and cohort studies. (I2 = 62%; *P*-value = 0.001) which could be due to the different study designs that were included in the analysis similar to previously published studies (56, 57). Regarding the outcome of PDA surgical ligation, meta-anaylsis of RCT studies revealed significantly lower rates of surgical PDA ligation among the infants given prophylactic doses of indomethacin (*P*-value < 0.001) which is similar to the findings of Fowlie et al. who reported a significant lower incidence of surgical PDA ligation among the indomethacin prophylactic group (typical RR 0.51, 95% CI 0.37,0.71) (14).

On the other hand, in the present study, no significant difference was reported between indomethacin prophylactic group and the placebo/no treatment group with regard to the outcome of BPD and pulmonary hemorrhage rates in the meta-analysis of cohort and RCT studies and combined analysis. Jensen et al. (57) in their analysis of observational data found that prophylactic indomethacin did not increase or decrease the risk of developing BPD. Moreover, the authors compared these results with another analysis of RCTs, however, the analysis indicated the same information that prophylactic indomethacin had no beneficial effects on BPD.

With regard to the risk of intraventricular hemorrhage, our analysis showed no significant difference between the group of infants given prophylactic indomethacin when compared to the placebo group. However, with regard to severe IVH, meta-analysis of RCT studies showed significantly lower rates of severe IVH in the prophylactic indomethacin group (*P*-value = 0.008). Similarly, Fowlie et al. found a significant reduction in severe IVH incidence in infants that were prophylactically injected with indomethacin (typical RR 0.66, 95% CI 0.53–0.82) (14). However, significant heterogeneity in this study was estimated due to the inconsistency of treatment efficacy among their included studies (56). None of the studies, however, measured the long-term outcomes, they have only focused on the short ones. Schmidt et al. (15) in their large trial on 18-month infants reported statistical insignificance on long term neurodevelopmental outcomes although IVH grade 3 and 4 were significantly reduced. Therefore, concerns should be made to assess the overall quality of the effect of indomethacin on the long-term neurodevelopmental outcomes and the rate of adverse events incidence due to the vasoconstrictive nature of the drug which may alter the cerebral blood flow.

Furthermore, we found no significance between the use of prophylactic indomethacin on infants in reducing the time of hospital stay. The findings reported by Fowlie et al. favored the control groups in terms of time spent in the hospital with no significance (*P* = 0.087) (14). With regard to the outcome of death, no significant effect of prophylactic indomethacin was reported in the current study in both cohort and RCT studies. Jensen et al. reported a weak association between indomethacin prophylaxis and decreased risk-adjusted odds of mortality (0.81, 95% CI 0.66–0.98), however, the authors included observational data only (57).

Limitations to our study include variable heterogeneity in the analysis of some outcomes due to the different study designs that were included in this study. However, we estimated the publication bias in most cases no publication bias was found.

## Conclusion

Prophylactic indomethacin in VLBW infants has proven efficient in preventing short-term events such as PDA, surgical PDA ligation, and severe IVH. On the other hand, it showed no significance with regard to outcomes such as IVH, BPD, pulmonary hemorrhage, necrotizing enterocolitis, intestinal performation, death and hospital stays. Since the meta-analysis results regarding effectiveness of prophylactic indomethacin varied based on the study design particularly with regard to outcomes such as surgical PDA ligation and severe IVH, this warrants the need for long term studies with larger sample size to determine the effectiveness of prophylactic indomethacin.

## Data Availability Statement

The raw data supporting the conclusions of this article will be made available by the authors, without undue reservation.

## Author Contributions

AAl proposed, conceptualized, and designed the study and wrote the manuscript. SA and AAS helped in the pilot extraction of a few manuscript for building the data extraction sheet and assessed the risk of bias among different included studies. All authors read and approved the final manuscript.

## Conflict of Interest

The authors declare that the research was conducted in the absence of any commercial or financial relationships that could be construed as a potential conflict of interest.

## Publisher’s Note

All claims expressed in this article are solely those of the authors and do not necessarily represent those of their affiliated organizations, or those of the publisher, the editors and the reviewers. Any product that may be evaluated in this article, or claim that may be made by its manufacturer, is not guaranteed or endorsed by the publisher.
